# [^18^F]CFT synthesis and binding to monoamine transporters in rats

**DOI:** 10.1186/2191-219X-2-3

**Published:** 2012-01-25

**Authors:** Sarita Forsback, Päivi Marjamäki, Olli Eskola, Jörgen Bergman, Johanna Rokka, Tove Grönroos, Merja Haaparanta, Olof Solin

**Affiliations:** 1 Radiopharmaceutical Chemistry Laboratory, Turku PET Centre, University of Turku, Porthaninkatu 3, Turku, 20500, Finland; 2 MediCity/PET Preclinical Imaging, Turku PET Centre, University of Turku, Tykistökatu 6A, Turku, 20520, Finland; 3 Accelerator Laboratory, Åbo Akademi University, Porthaninkatu 3, Turku, 20500, Finland

**Keywords:** [^18^F]CFT, DAT, NET, electrophilic fluorination, monoamine transporters

## Abstract

**Background:**

We present the electrophilic synthesis of [^18^F]2β-carbomethoxy-3β-(4-fluoro)tropane [[^18^F]CFT] and the pharmacological specificity and selectivity of [^18^F]CFT for monoamine transporters in the brain and peripheral organs of rats. The human radiation dose is extrapolated from the animal data.

**Methods:**

[^18^F]CFT was synthesized by electrophilic fluorination of a stannylated precursor by using post-target-produced [^18^F]F_2 _as a fluorinating agent. The *ex vivo *^18^F-activity biodistribution of [^18^F]CFT in the brain of rats was studied by autoradiography. The binding of [^18^F]CFT to the monoamine transporters was studied using *in vivo *blocking experiments with dopamine transporter [DAT], norepinephrine transporter [NET], or serotonin transporter [SERT] inhibitors. *In vivo *animal positron emission tomography was used as a comparative method to determine tracer kinetics. Human radiation dose was assessed using OLINDA software.

**Results:**

The radiochemical yield of [^18^F]CFT from the initial [^18^F]F^-^, decay corrected to the end of bombardment, was 3.2 ± 1.0%. The specific activity [SA] was 14.5 ± 3.4 GBq/μmol, decay corrected to the end of synthesis. Radiochemical purity exceeded 99%. DAT-specific binding was found in the striatum, locus coeruleus, and pancreas. NET-specific binding was found in the locus coeruleus. SERT-specific binding was not found in any of the studied organs. Effective dose equivalent [EDE] estimated for the standard human model was 12.8 μSv/MBq. Effective dose [ED] was 9.17 μSv/MBq.

**Conclusions:**

Post-target-produced high-SA [^18^F]F_2 _was used to incorporate^18^F directly into the phenyl ring of [^18^F]CFT. The final product had high radiochemical and chemical purities and a high SA for DAT and NET studies *in vivo*. In periphery, [^18^F]CFT showed a specific uptake in the pancreas. EDE and ED corresponded well with other^18^F-radioligands.

## Background

Dopamine transporters [DAT] are proteins located in the dopaminergic nerve terminals; they regulate the synaptic concentration of dopamine in the brain. Changes in the density and function of DAT in the brain are involved in many neurodegenerative and neuropsychiatric disorders, such as Parkinson's disease and schizophrenia. These changes can be imaged using positron emission tomography [PET].

Many radioligands, including [^11^C]CFT [1] and several [^18^F]F-labeled phenyl tropane analogs of cocaine [2], have been used to study dopamine reuptake in living subjects. However, none of these fulfill the requirements for an optimal radioligand for DAT imaging. [^11^C]CFT suffers from slow kinetics compared with the short half-life of^11^C (*T*_1/2 _= 20.4 min). The phenyl tropane analogs have a high or moderate affinity with other monoamine transporters (i.e., serotonin transporters [SERT] and norepinephrine transporters [NET]), or they undergo extensive metabolism. More recently, the new [^18^F]F-labeled phenyl tropane analog [^18^F]FE-PE2I has shown promise as a radioligand for DAT [3], despite its relatively fast metabolism [4].

**Figure 1:**
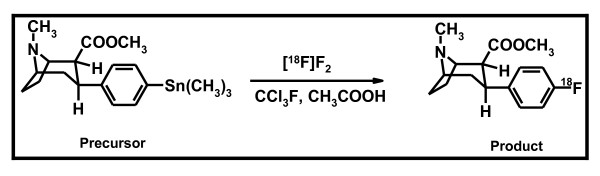
**Scheme depicting the use of a stannylated *precursor *to synthesize [^18^F]CFT *product***.

Previously, electrophilic fluorination of a stannylated precursor, 2β-carbomethoxy-3β-(4-trimethylstannylphenyl)tropane (*precursor*) to achieve 2β-carbomethoxy-3β-(4-[^18^F]-fluorophenyl)tropane [[^18^F]CFT] (*product*) (see Figure 1) and preliminary evaluation of the radioligand in rats were reported by Haaparanta et al. [5] and by Bergman et al. [6]. A report on the ability of [^18^F]CFT to reflect nigral dopaminergic cell loss in a rat model of Parkinson's disease [7] as well as a study comparing the brain accumulation, metabolism, and kinetics of [^18^F]CFT and [^18^F]CFT-FP [8] have shown that [^18^F]CFT can be used to image DAT in rats. The suitability of [^18^F]CFT as a radioligand for *in vivo *studies of DAT in humans has been evaluated [9], and [^18^F]CFT has been used in human studies of Parkinson's disease [10-15], schizophrenia [16,17], and detached personality [18]. [^18^F]CFT was proven to be a suitable radiotracer to image DAT by PET in humans due to its high target-to-nontarget ratio and low metabolism [9] although [^3^H]CFT has also been shown to have some affinity to SERT and NET [19,20]. The kinetics of [^18^F]CFT are relatively slow, but the half-life of^18^F (*T*_1/2 _= 109.8 min) allows equilibrium between specific and nonspecific binding during a human PET study.

A PET radioligand suitable for DAT must have a moderate to high specific activity [SA] to avoid saturation of transporter sites (with associated pharmacological effects) in patients. High SA can be easily achieved by nucleophilic fluorination. The^18^F label is usually incorporated into a molecule via an alkyl side chain, as in the case of [^18^F]FE-PE2I [3] or [^18^F]CFT-FP [21]. However, side chains are often prone to fast metabolism. A more metabolically stable configuration can be achieved by inserting^18^F directly into the phenyl ring via electrophilic fluorination. [^18^F]F_2 _is traditionally produced by either^20^Ne(d,α)^18^F with an added F_2 _carrier [22] or^18^O(p, n)^18^F using^18^O_2_/F_2 _as target [23]. The latter method is more efficient than the former [24]. However, both production methods suffer from low SA. Post-target-produced [^18^F]F_2 _has 100- to 1,000-fold higher SA than the traditional methods [25]. Therefore, post-target-produced [^18^F]F_2 _offers the possibility of producing high-affinity radioligands through electrophilic labeling.

In the present study, we report the optimized electrophilic synthesis of [^18^F]CFT with high SA and its quality assurance for clinical PET studies. The pharmacological specificity and selectivity of [^18^F]CFT for monoamine transporters *ex vivo *are reported. The distribution of^18^F activity in the brain and peripheral organs of rats is reported *ex vivo *and *in vivo *in order to compare the methods in the determination of tracer kinetics. The human radiation dose is extrapolated from the animal data using organ level internal dose assessment [OLINDA]/EXM 1.0 software (OLINDA EXM, Vanderbilt University, Nashville, TN, USA) [26].

## Methods

### Chemicals and equipment

The stannylated *precursor *and the reference compound, 2β-carbomethoxy-3β-(4-fluorophenyl)tropane, were supplied by ABX (ABX GmbH, Radeberg, Germany). All other reagents that were purchased from commercial suppliers were either of synthesis grade or analytical grade and were used without further purification.

Semi-preparative high-performance liquid chromatography [HPLC] was performed using a Merck-Hitachi L-6200 HPLC pump (Merck AG, Darmstadt, Germany) and a Waters μBondapak C18 column (7.8 × 300 mm, 10 μm; Waters Corporation, Milford, MA, USA). A Merck-Hitachi L-7400 UV-absorption detector (*λ *= 215 nm) and a 2 × 2-in NaI crystal for^18^F-activity detection were used. The column was eluted with 0.01 M H_3_PO_4_/CH_3_CN (7:3; flow rate of 3 ml/min).

Analytical HPLC was conducted using a Merck-Hitachi L-7100 HPLC pump, an Atlantis dc18 column (5 μm; Waters Corporation, Milford, MA, USA), a Merck-Hitachi L-7400 UV-absorption detector (*λ *= 215 nm) and a 2 × 2-in NaI crystal for^18^F-activity detection. The eluent used was 0.01 M H_3_PO_4_/CH_3_CN (75:25; flow rate 1.1 ml/min).

Liquid chromatography/mass spectrometry [LC/MS] was performed with a PE SCIEX API 150 EX mass spectrometer (PerkinElmer SCIEX, Toronto, Canada) equipped with a turbo ion-spray source, a PerkinElmer series 200 micro pump (PerkinElmer Instruments, Branford, CT, USA), and a Waters Symmetry C18 column (2.1 × 30 mm, 3.5 μm; Waters Corporation, Milford, MA, USA) were used to measure the concentration of CFT. The column was eluted with MeOH/0.2% HCOOH(aq) (flow rate 0.1 ml). A Supor Acrodisc (0.2 μm, 13 mm; Pall Corporation, NY, USA) sterile filter was used to formulate [^18^F]CFT for injection.

### Production of [^18^F]F^-^

[^18^F]F^- ^was obtained via the nuclear reaction^18^O(p, n)^18^F by irradiating 700 μl^18^O-enriched water with a 17-MeV proton beam produced by an MGC-20 cyclotron (Efremov Institute of Electrophysical Apparatuses, St Petersburg, Russia).

### Production of [^18^F]F_2_

[^18^F]F_2 _was synthesized in an electrical discharge chamber by the^18^F/^19^F exchange reaction. The^18^F source was [^18^F]fluoromethane, which was mixed with a low amount (290 to 400 nmol) of carrier fluorine in neon (Ne/0.5% F_2_) inside the discharge chamber. [^18^F]Fluoromethane was produced from methyl iodide by a nucleophilic substitution reaction with a [^18^F]F^-^/Kryptofix K2.2.2 complex in acetonitrile. A detailed description of the [^18^F]F_2 _synthesis is presented elsewhere [25].

### Synthesis of [^18^F]CFT

The stannylated precursor (*precursor*, 250 to 500 μg, 0.6 to 1.2 μmol) was dissolved in a mixture of trichlorofluoromethane (Freon-11, 600 μl) and dry acetic acid (100 μl). [^18^F]F_2 _was bubbled through this mixture at room temperature. Freon-11 was evaporated using neon flow, and 600 μl of preparative HPLC eluent was added to the residue. With no further modifications, this solution was then loaded onto the preparative HPLC column.

**Figure 2:**
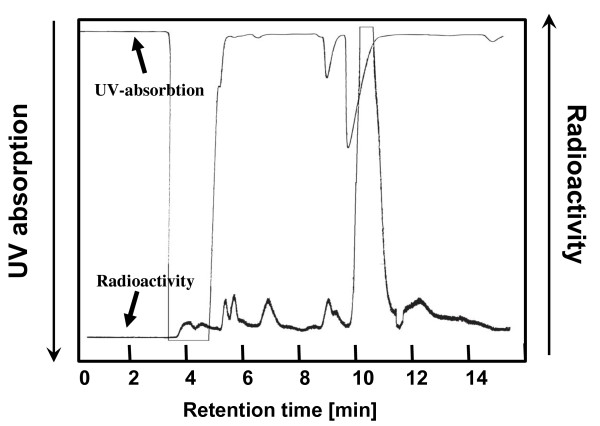
**Sample chromatogram of a semi-preparative HPLC separation of [^18^F]CFT from the reaction mixture**.

[^18^F]CFT was purified by semi-preparative HPLC using the system described in the 'Chemicals and equipment' section. The 3-ml fraction containing the [^18^F]CFT was collected (Figure 2). The fraction was evaporated to dryness with a vacuum evaporator, formulated into a 0.9% NaCl/0.1 M phosphate buffer (phosphate buffer pH 7, 3:2, *v*/*v*), and passed through the sterile filter into the end product vial.

### Quality of [^18^F]CFT

The^18^F activity, pH, and volume were measured from the end product. A sample from the end product was evaluated by the analytical HPLC system described in the 'Chemicals and equipment' section. Determinations of chemical purity, radiochemical purity [RCP], and SA were conducted by comparing HPLC retention times and peak intensities with a reference compound of known concentration and^18^F-activity concentration. Radiochemical yields [RCY] were calculated from the initial amount of [^18^F]F^- ^and decay corrected to the end of bombardment [EOB]. The SA of the product was decay corrected to the end of the semi-preparative HPLC separation [EOS].

The SA of the final product was also determined with LC/MS by measuring the [^19^F]CFT mass concentration using the same reference as was used with analytical HPLC. The analyses were performed in positive selected ion monitoring mode for *m*/*z *= 278 (corresponding to the protonated molecule [MH^+^] of [^19^F]CFT), and the SAs are decay corrected to the EOS.

### Animals

Sprague-Dawley rats (Harlan Sprague-Dawley, Indianapolis, IN, USA) weighing 251 ± 59 g (15 females, 20 males) were used in this study. The rats were housed under standard conditions (temperature 21°C; relative humidity 55 ± 5%; 12-h light/dark cycle) with free access to tap water and standard food. Animal care was in accordance with the guidelines of the International Council of Laboratory Animal Science. The Turku University ethics committee for animal experiments and the Animal Experiment Board of the Province of Southern Finland approved this study.

### Biodistribution studies

[^18^F]CFT in 0.9% NaCl/0.1 M phosphate buffer (3:2, *v*/*v*, pH 7) was injected into the tail vein of rats that are sedated with CO_2_/O_2 _(50:50 vol.%). The^18^F activity injected via [^18^F]CFT per rat was 57 ± 24 MBq (range, 12 to 181 MBq). This corresponds to a 23 ± 10-nmol/kg (5 to 40 nmol/kg) administration of CFT, as calculated from the SA that was determined using the HPLC method at the time of injection. The animals were killed in a CO_2 _chamber at 10 min (*n *= 2), 20 min (*n *= 3), 40 min (*n *= 6), or 120 min (*n *= 3) after injection of the tracer. The brains were rapidly removed, and a piece from the cerebellar cortex of each brain was dissected, measured for^18^F activity in a calibrated 3 × 3-in NaI(Tl) well counter (Bicron, Newbury, OH, USA), and weighed. After decay correction, these data, expressed as the percentage of injected dose per gram of tissue [%ID/g], were used to calibrate the absolute uptake of^18^F activity in autoradiographic brain images. The rest of the brain was frozen in isopentane, chilled with dry ice for sectioning with a cryomicrotome, and handled as described in the 'Digital autoradiography' section.

Other organs and tissue samples were rapidly dissected, weighed, and measured for^18^F activity. The decay-corrected uptake of^18^F activity in the organs and tissues was expressed as %ID/g.

### Pharmacological studies

The specificity of [^18^F]CFT binding to DAT in the brain in pretreated rats was assessed with a selective DAT antagonist, GBR12909 (5 mg/kg, Sigma-RBI, St. Louis, MO, USA). Selectivity was examined by injecting rats with a 5-mg/kg dose of fluoxetine, a selective antagonist for SERT (Sigma-RBI, St. Louis, MO, USA), or with a 5-mg/kg dose of nisoxetine, a selective antagonist for NET (RBI, Natick, MA, USA). Binding profiles of the antagonists are presented in Table 1. GBR12909, fluoxetine, or nisoxetine dissolved in distilled H_2_O/0.9% NaCl (50:50, *v*/*v*, 2 mg/ml) were injected intravenously into rats 60 min prior to the injection of [^18^F]CFT. The rats were killed by CO_2 _inhalation 40 min after injection of [^18^F]CFT. The brains and organs were handled as in the biodistribution studies. The regional distribution of^18^F activity in the brains of control rats (*n *= 6) and in the brains of rats that were pretreated with GBR12909 (*n *= 7), fluoxetine (*n *= 6), or nisoxetine (*n *= 6) were determined using digital autoradiography.

**Table 1:** Binding profiles of the monoamine transporter antagonist used in this study

Antagonist	DAT	SERT	NET
CFT [37]	22.9 ± 0.4	100 ± 13	38.6 ± 9.9
GBR12909 [38]	10.6 ± 1.9^a^	132 ± 0^b^	496 ± 22^c^
Fluoxetine [39]	3600 ± 100	0.81 ± 0.02	240 ± 10
Nisoxetine [40]	477	383	5.1

^a^Half maximum inhibitory concentration [IC_50_] (nanomolar [nM]) of [^3^H]CFT;^b^IC_50 _(nM) of [^3^H]citalopram;^c^IC_50 _(nM) of [^3^H]nisoxetine. DAT, dopamine transporter; SERT, serotonin transporter; NET, norepinephrine transporter. Values are Ki (nM) ± SEM, unless otherwise stated.

### Digital autoradiography

Coronal brain sections (20 μm) were thaw-mounted onto microscope slides, air dried, and apposed to an imaging plate (Fuji Imaging Plate BAS-TR2025, Fuji Photo Film Co., Ltd., Minato-ku, Tokyo, Japan) for 4 h. The imaging plates were scanned with the Fuji Analyzer BAS-5000.

The digital autoradiographic images were analyzed for count density (photo-stimulated luminescence per unit area [PSL/mm^2^]) with a computerized image analysis program (Tina 2.1, Raytest Isotopenmessgeräte, GmbH, Straubenhardt, Germany). Regions of interest [ROIs] were drawn over the frontal cortex, striatum, locus coeruleus, and cerebellum, which were anatomically identified from the cryomicrotome sections using a rat brain atlas [27]. At least 10 sections were analyzed for each brain region, and the count densities for background areas were subtracted from the image data. PSL/mm^2 ^values were converted into %ID/g values as previously described in the 'Biodistribution studies' section.

### PET imaging

Two PET scans were carried out using an Inveon multimodality PET/computed tomography [CT] (Siemens Medical Solutions, Knoxville, TN, USA) designed for rodents and other small laboratory animals. The device provides 159 transaxial slices, a 10.0-cm transaxial field of view [FOV], and a 12.7-cm axial FOV. Rats were anesthetized with 2% isoflurane approximately 15 min before measurements. The body temperature of each rat was maintained with a heating pad on which the rat lies. Following the transmission scan for attenuation correction using the CT modality, an emission scan was acquired for 120 min in three-dimensional [3-D] list mode with an energy window of 350 to 650 keV. The scans started immediately after intravenous injection of [^18^F]CFT (dose 27.9 MBq, mass 830 ng, SA 9.3 GBq/μmol and dose 38.0 MBq, mass 1,200 ng, SA 8.8 GBq/μmol at time of injection, respectively). List mode data were stored in 3-D sinograms, which were then Fourier-rebinned into two-dimensional [2-D] sinograms (45 frames with dimensions of 20 × 15 s, 15 × 600 s, 10 × 600 s). The image was reconstructed using 2-D-filtered back projection with a 0.5-mm RAMP filter. ROIs were placed on the striatum, cerebellar cortex, frontal cortex, and liver using the Inveon Research Workplace Image Analysis software (Siemens Medical Solutions USA, Knoxville, TN, USA) and with a CT template as an anatomical reference.

### Dosimetry

The animal %ID/g tissue data was extrapolated to humans using the percentage kilogram per gram method [28]. In this method, the animal %ID/g value is first multiplied with the animal's weight and then multiplied with the human organ weight/human weight ratio. Human radiation dose was estimated from these values using OLINDA/EXM 1.0 software [26].

### Data analysis and statistical procedures

Statistical analyses were performed using the SPSS Statistics 17.0 software (SPSS Inc., Chicago, IL, USA). Means were considered significantly different when *p *< 0.05. Comparison of SAs was tested using Student's *t *test (paired, two samples for mean assuming unequal variances).

Effects of the pretreatments were tested using repeated measurement analysis of variance. Results are expressed as means ± SD for the indicated number of observations.

## Results

### Synthesis and quality of [^18^F]CFT

Electrophilic fluorination was applied to a stannylated *precursor *(Figure 1) to synthesize [^18^F]CFT *product *(*n *= 24). The initial [^18^F]F^- ^activity was 37 ± 3 GBq (range, 32 to 42 GBq), and the average synthesis time was 43 ± 3 min, including the synthesis of [^18^F]F_2_, radiofluorination, and semi-preparative purification. In a semi-preparative HPLC purification, the [^18^F]CFT fraction eluting at 10.5 min was collected (Figure 2). Evaporation to dryness and formulation for injection took an additional 10 min.

**Figure 3:**
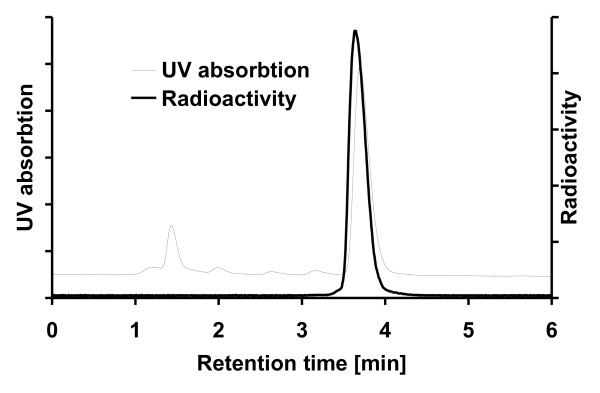
**Sample chromatogram of radio-HPLC analysis of formulated [^18^F]CFT**.

The RCY calculated from initial [^18^F]F^- ^(decay corrected to EOB) was 3.2 ± 1.0%, and^18^F activity of [^18^F]CFT was 917 ± 278 MBq (501 to 1,395 MBq) at EOS. The SA measured by analytical HPLC was 14.5 ± 3.4 GBq/μmol (8.9 to 23.6 GBq/μmol with all values decay corrected to EOS). From analytical HPLC studies (Figure 3), the RCP exceeded 99% in all cases. The pH of the final product was 7. The final product was radiochemically stable for up to 6 h.

From selected batches (*n *= 19), the SA of the final product was determined by LC/MS. The SA of these batches measured by analytical HPLC was 14.9 ± 3.1 GBq/μmol. The SA measured by LC/MS was 18.2 ± 5.9 GBq/μmol. SAs calculated using the analytical HPLC method differed significantly from the SAs calculated using the LC/MS method (*p *= 0.04).

### Biodistribution and pharmacological studies

**Figure 4:**
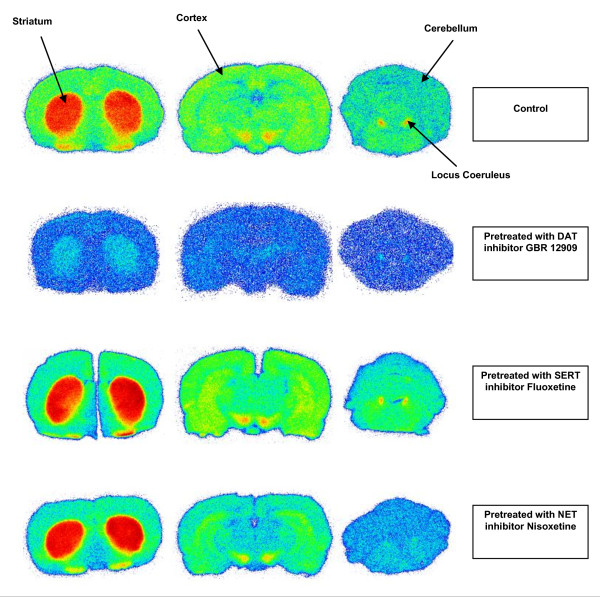
**Representative [^18^F]CFT autoradiographic images**. Brain slices from control rats and from rats pretreated with GBR12909, fluoxetine, or nisoxetine.

The^18^F-activity accumulation in the striatum, locus coeruleus, frontal cortex, and cerebellum of control rats and monoamine inhibitor-pretreated rats 40 min after [^18^F]CFT injection is presented in Table 2. Pretreatment with GBR12909 significantly reduced the [^18^F]CFT uptake in the striatum (*p *= 0.006) and locus coeruleus (*p *= 0.02). In nisoxetine-pretreated rats, the [^18^F]CFT uptake decreased significantly in the locus coeruleus (*p *< 0.005). Fluoxetine pretreatment had no effect on the accumulation of the^18^F activity in any region studied. Autoradiograms of representative brain sections from a control rat and from rats pretreated with GBR12909, fluoxetine, or nisoxetine are shown in Figure 4.

**Table 2:** ^18^F-activity uptake 40 min after injection of [^18^F]CFT into the brains of control and pretreated rats

Brain region	Control *n *= 6	GBR12909 *n *= 7	Nisoxetine *n *= 6	Fluoxetine *n *= 6
Striatum	1.55 ± 0.78	0.49 ± 0.18*	1.48 ± 0.22	1.38 ± 0.75
Locus coeruleus	0.67 ± 0.29	0.37 ± 0.11**	0.23 ± 0.05*	0.47 ± 0.16
Frontal cortex	0.32 ± 0.08	0.23 ± 0.04	0.26 ± 0.03	0.33 ± 0.13
Cerebellum	0.20 ± 0.04	0.15 ± 0.04	0.17 ± 0.02	0.23 ± 0.09

*p < 0.01 compared with control;**p < 0.05 compared with control. Uptake values are in percent injected dose per gram tissue. All values are means ± SD. The effect of pretreatment was compared with the uptake values of control rats. Means were considered significantly different when *p *< 0.05.

**Figure 5:**
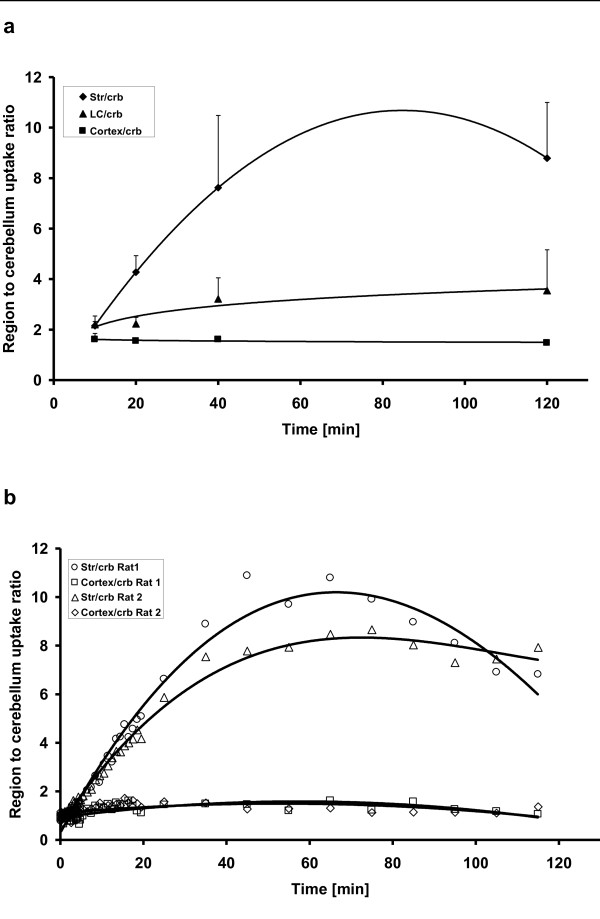
**The time courses of the brain region-to-cerebellum ratios**. They are from the *ex vivo *study after [^18^F]CFT injection where *n *= 2 to 4/time point (**a**) and from the *in vivo *animal PET study where *n *= 2 (**b**).

The region-to-cerebellum ratios at different time points from the *ex vivo *and *in vivo *studies are shown in Figure 5a,b, respectively. In *ex vivo *studies, the striatum-to-cerebellum ratio increased from 2.1 ± 0.2 at 10 min to 8.8 ± 2.2 at 120 min. The locus coeruleus-to-cerebellum ratio was 2.2 ± 0.3 at 10 min and 3.5 ± 1.6 at 120 min. The frontal cortex-to-cerebellum ratio was constant, ranging from 1.4 to 1.6 at all time points and with all pretreatments. All monoamine inhibitors used in this study significantly decreased the locus coeruleus-to-cerebellum ratio. Pretreatment with GBR12909 significantly reduced the striatum-to-cerebellum ratio.

**Figure 6:**
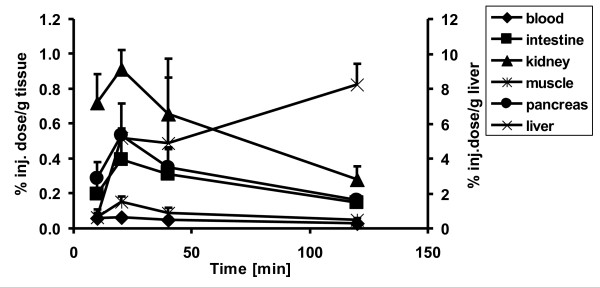
**Time course of the [^18^F]CFT uptake (percent injected dose per gram tissue) by selected organs**. The uptake values for the liver are presented in the *y*-axis on the right.

The^18^F-activity accumulation in the peripheral organs and tissues after the injection of [^18^F]CFT is presented in Table 3. The accumulation of [^18^F]CFT-derived^18^F activity peaked at 20 min in most tissues and decreased thereafter (Figure 6). High levels of^18^F activity were recorded in the liver, kidneys, and spleen. In the liver, the highest uptake (8.3 ± 1.2% ID/g) was measured 120 min after injection of [^18^F]CFT.^18^F-activity accumulation in the bone increased slowly with time, but it was still low (0.19 ± 0.15% ID/g) at 120 min.

**Table 3:** ^18^F-activity uptake after injection of [^18^F]CFT in the organs of control and pretreated rats

Organ	10 min *n *= 2	20 min *n *= 3	40 min *n *= 4	120 min *n *= 3	GBR12909 *n *= 3	Nisoxetine *n *= 3	Fluoxetine *n *= 2
Blood	0.06 ± 0.03	0.07 ± 0.01	0.05 ± 0.02	0.03 ± 0.01	0.04 ± 0.01	0.06 ± 0.01	0.03 ± 0.02
Liver	0.65 ± 0.45	5.17 ± 1.99	4.90 ± 3.74	8.26 ± 1.16	3.38 ± 0.94	5.86 ± 0.10	4.22 ± 1.22
Adrenal gland	0.28 ± 0.14	0.36 ± 0.01	0.24 ± 0.03	0.17 ± 0.06	0.23 ± 0.07	0.25 ± 0.02	0.24 ± 0.11
Spleen	0.36 ± 0.24	0.88 ± 0.28	0.49 ± 0.05	0.26 ± 0.13	0.50 ± 0.19	0.44 ± 0.07	0.44 ± 0.02
Pancreas	0.28 ± 0.10	0.53 ± 0.04	0.35 ± 0.11	0.16 ± 0.01	0.17 ± 0.05*	0.31 ± 0.02	0.22 ± 0.12
Kidney	0.72 ± 0.16	0.91 ± 0.11	0.66 ± 0.31	0.28 ± 0.07	0.53 ± 0.13	0.47 ± 0.02	0.41 ± 0.18
Stomach	0.12 ± 0.09	0.53 ± 0.38	0.19 ± 0.05	0.08 ± 0.05	0.47 ± 0.37	0.11 ± 0.01	0.17
Lung	0.51 ± 0.37	0.46 ± 0.07	0.29 ± 0.08	0.17 ± 0.06	0.20 ± 0.05	0.25 ± 0.01	0.25 ± 0.04
Heart	0.14 ± 0.06	0.16 ± 0.03	0.11 ± 0.02	0.07 ± 0.02	0.10 ± 0.03	0.11 ± 0.01	0.13 ± 0.02
Muscle	0.07 ± 0.02	0.15 ± 0.03	0.09 ± 0.03	0.05 ± 0.01	0.08 ± 0.02	0.10 ± 0.01	0.07 ± 0.03
Urinary bladder	0.14 ± 0.14	0.51 ± 0.23	0.31 ± 0.13	0.24 ± 0.13	0.30 ± 0.12	0.28 ± 0.01	0.22 ± 0.04
Bone	0.03 ± 0.01	0.09 ± 0.02	0.07 ± 0.04	0.19 ± 0.15	0.06 ± 0.01	0.31 ± 0.20	0.09 ± 0.04
Marrow	0.22 ± 0.16	0.63 ± 0.17	0.47 ± 0.10	0.20 ± 0.05	0.39 ± 0.12	0.49 ± 0.05	0.40 ± 0.04
Fat, subcutaneous	0.03 ± 0.01	0.06 ± 0.01	0.07 ± 0.03	0.03 ± 0.01	0.04 ± 0.02	0.07 ± 0.01	0.05 ± 0.03

*p < 0.05 compared to 40 min. Uptakes (percent injected dose per gram tissue) occurred in the organs of control rats at different time points and in organs of pretreated rats at 40 min. All values are means ± SD. The effect of pretreatment was compared with the uptake values of control rats at 40 min. Means were considered significantly different when *p *< 0.05.

Pretreatment of the rats with GBR12909 significantly decreased the uptake of^18^F activity in the pancreas (*p *= 0.03). No significant changes in the^18^F-activity uptake were recorded in the periphery of the rats pretreated with nisoxetine or fluoxetine.

### PET imaging

**Figure 7:**
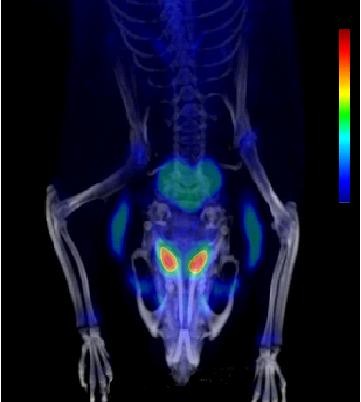
**Animal PET-CT image of [^18^F]CFT accumulation in the brain of rats**. Data were collected for 2 to 120 min post injection.

The *in vivo *distribution and uptake of^18^F activity after [^18^F]CFT injection in the brain of rats are presented in Figure 7. The uptake of^18^F activity in the striatum and cerebellum peaked during the first 5 to 10 min and decreased thereafter. The striatum-to-cerebellum ratio reached the maximum value of approximately 9 at 60 min. The time courses of the striatum-to-cortex and cortex-to-cerebellum ratios are shown in Figure 5b. The highest *in vivo *uptake of^18^F activity in the periphery was observed in the liver. The uptake increased in the liver during the first 60 min of PET imaging and was almost constant until the end of the scanning (i.e., until 120 min after injection).

### Dosimetry

The effective dose equivalent [EDE] estimated for the standard human model was 12.8 μSv/MBq. Effective dose [ED] was 9.17 μSv/MBq.

## Discussion

Post-target-produced [^18^F]F_2 _[25] offers a feasible method to produce PET tracers with high SA for neuroimaging through electrophilic fluorination. When using post-target-produced [^18^F]F_2_, the SA depends on several factors, such as the initial amount of [^18^F]F^- ^and the amount of carrier fluorine used in the^19^F-^18^F exchange reaction [25]. The SA of [^18^F]CFT can potentially be increased by further optimizing this^19^F-^18^F exchange reaction by decreasing the amount of carrier F_2_. However, in our hands, this resulted in a dramatic decrease in RCY. The amount of carrier F_2 _used in this study (290 to 400 nmol) is a compromise, offering SA and RCY that are high enough for several human PET studies from one production run. The radiochemical and chemical purities of the final product were verified by HPLC, and both were found to fulfill the requirements for human injection (i.e., RCP > 95.0% and the absence of unknown signals in the UV trace; Figure 3). Signals from compounds other than CFT observed in the UV trace were from the formulation solution.

For radioligands having very high SA, the sensitivity limitation of UV detection means that LC/MS can be the only method to determine the SA [29]. In the present case where [^18^F]CFT is synthesized through electrophilic fluorination at high SA, we therefore compared the HPLC/UV absorption and the LC/MS technique for the determination of SA. In quantitative analyses, LC/MS is a faster and more sensitive method than HPLC combined with a UV detector. However, LC/MS is more easily affected by changes in the sample matrix. In this study, the SAs were significantly higher when determined by the LC/MS method than by the HPLC method (i.e., the concentration of CFT was lower when measured by LC/MS than by HPLC). This could be due to ion suppression in LC/MS [30]. The effect of ion suppression could be diminished by more extensive chromatographic separation or sample preparation prior to MS. However, both methods of analysis are suitable for analysis in the present case.

In the biodistribution study, the highest level of^18^F activity was found in the main excretory organs. Additionally, the uptake in the bone was low even at 120 min, reflecting the good stability of the carbon-fluorine bond.^18^F activity accumulated in the liver with the highest value at 120 min, indicating slow excretion and low metabolism. In all other organs studied, the^18^F activity peaked 20 min after injection (see Table 3). The results from the *in vivo *study were in accordance with the *ex vivo *findings. [^18^F]CFT has been reported to be relatively resistant to metabolism; in a microdialysis study of 120 min in rodents, the amount of unmetabolized [^18^F]CFT was approximately 64% of the total^18^F activity [31].

In the periphery, non-neuronal DAT expression and DAT immunoreactivity have been found in the stomach, pancreas, and kidneys [32]. [^18^F]CFT uptake in the pancreas, stomach, and kidneys was moderate in the present study. After GBR12909 pretreatment, the^18^F-activity uptake decreased significantly in the pancreas, indicating DAT-specific binding. In this study, no NET- or SERT-specific binding of^18^F activity was found in the periphery even though extraneuronal NET expression has been found in the lungs, adrenal medulla, and placenta [32].

In the brain, the accumulation of^18^F activity in the striatum was specific for DAT; it was significantly decreased with GBR12909 pretreatment (Figure 4). In addition to DAT, the striatum contains a low density of SERT, and NET is virtually absent [33]. Neither fluoxetine nor nisoxetine pretreatment affected the^18^F-activity uptake in the striatum. High^18^F-activity accumulation was seen in the locus coeruleus, a brain region with high NET density as has been earlier shown by Burchett et al. [34]. A similar finding in monkeys using [^3^H]CFT was observed by Kaufman and Madras [19]. The^18^F-activity accumulation in locus coeruleus was significantly decreased in rats pretreated with nisoxetine, which indicates that NET sites also bind with [^18^F]CFT. Because NET is virtually absent in the striatum, [^18^F]CFT is suitable for imaging striatal DAT sites. However, the accumulation of^18^F activity in locus coeruleus was also significantly decreased in rats pretreated with GBR12909. The affinity of CFT for DAT and NET is of the same order of magnitude (Table 1). With the relatively high dose of GBR12909 used in the pretreatment of the animals, it is evident that although the affinity of GBR12909 for NET is 50-fold less than for DAT, this dosage is high enough to displace [^18^F]CFT from the NET sites in the locus coeruleus. It is noteworthy that the *p *value in the statistical analyses is higher for the locus coeruleus than for the striatum in the GBR12909 blocking study.

In the *ex vivo *study, the uptake ratios for the striatum, locus coeruleus, and cortex versus the cerebellum reached a maximum between 40 and 120 min. In the *in vivo *study, the maximum striatum-to-cerebellum ratio was reached at 60 min. In both studies, the absolute values for this ratio were similar in the range of 9 to 10. It is noteworthy that due to its small size, the locus coeruleus cannot be analyzed from the *in vivo *PET study. These parallel studies provide a good demonstration of the strengths and weaknesses of different methods in radiopharmacological studies. Overall, the distribution of^18^F activity in rats after [^18^F]CFT injection was in good agreement with our earlier preliminary studies [5,8] and with studies using [^3^H]CFT [19,35].

The human ED and EDE values for [^18^F]CFT are 9.17 μSv/MBq and 12.8 μSv/MBq, respectively, and they are in line with those of another dopamine transporter ligand,^18^F-FPCIT [36]. Extrapolation of the animal data to humans to estimate the human radiation dose is inexact, but the order of magnitude of the EDE and ED values for [^18^F]CFT correspond well with those of other^18^F-labeled radioligands.

## Conclusions

Post-target-produced high-SA [^18^F]F_2 _was used to incorporate^18^F directly into the phenyl ring of [^18^F]CFT. The final product had high radiochemical and chemical purities and a high SA for neurotransmitter studies *in vivo*. It is noteworthy that as [^18^F]CFT shows a specific binding to NET in addition to DAT, [^18^F]CFT can also be used for imaging NET. The finding that [^18^F]CFT shows specific uptake in the pancreas also warrants future studies in humans with respect to potential utility in pancreatic imaging.

## Competing interests

The authors declare that they have no competing interests.

## Authors' contributions

SF is the first author and has taken part in all aspects of preparing the manuscript. PM has contributed in the concept and design of the study, in acquiring preclinical data, and in analyzing and interpreting this set of data, as well as in drafting the manuscript. OE has contributed in the acquisition of radiochemical data and analyzing and interpreting this set of data, as well as in drafting the manuscript. JB has contributed in the concept and design of the study and has enhanced the intellectual content of the manuscript. JR has contributed in acquiring the dosimetric data, in analyzing and interpreting this set of data, and in drafting the manuscript. TG has contributed in acquiring the preclinical data, in analyzing and interpreting this set of data, and in drafting the manuscript. MH has contributed in the concept and design of the study, in acquiring data, in analyzing and interpreting data, and in drafting the manuscript and has critically contributed to and revised the manuscript, as well as approved the final content of the manuscript. OS has contributed in the concept and design of the study, in analyzing and interpreting data, and in drafting the manuscript, as well as in approving the final content of the manuscript. All authors have read and approved the final manuscript.
